# Two decades of SVT ablation in Denmark: a trend towards higher age, more comorbidity, and less prior use of antiarrhythmic and rate-limiting pharmacotherapy—a nationwide registry-based Danish study

**DOI:** 10.1007/s10840-023-01692-9

**Published:** 2023-12-18

**Authors:** Charlotte Middelfart, Jacob Tønnesen, Christopher R. Zörner, Lise Da Riis-Vestergaard, Maria Hang Xuan Pham, Jannik Langtved Pallisgaard, Martin H. Ruwald, Peter Vibe Rasmussen, Arne Johannessen, Jim Hansen, Rene Worck, Gunnar Gislason, Morten Lock Hansen

**Affiliations:** 1 Department of Cardiology, Copenhagen Cardiovascular Research Center, Copenhagen University Hospital Herlev and Gentofte, Hellerup, Denmark; 2 Department of Clinical Medicine, Faculty of Health and Medical Sciences, University of Copenhagen, Copenhagen, Denmark; 3 The Danish Heart Foundation, Copenhagen, Denmark; 4 The National Institute of Public Health, University of Southern Denmark, Copenhagen, Denmark

**Keywords:** Supraventricular tachycardia, Catheter ablation, Patient characteristics, Antiarrhythmic therapy

## Abstract

**Background and aims:**

Trends in patient selection and use of pharmacotherapy prior to catheter ablation (CA) for supraventricular tachycardia (SVT) are not well described. This study examined temporal trends in patients undergoing first-time CA for regular SVT, including atrioventricular nodal re-entry tachycardia (AVNRT), accessory pathways (APs), and ectopic atrial tachycardia (EAT) on a nationwide scale in Denmark in the period 2001–2018.

**Methods and results:**

Using Danish Nationwide registers, 9959 patients treated with first-time CA for SVT between 2001 and 2018 were identified, of which 6023 (61%) received CA for AVNRT, 2829 (28%) for AP, and 1107 (11%) for EAT. Median age was 55, 42, and 55 in the AVNRT, APs, and EAT group, respectively. The number of patients receiving CA increased from 1195 between 2001 and 2003 to 1914 between 2016 and 2018. The percentage of patients with a CHA_2_DS_2_-VASc score ≥ 2 increased in all patient groups. The number of patients who underwent CA with no prior use of antiarrhythmic- or rate limiting medicine increased significantly, though prior use of beta-blockers increased for AVNRT patients. Use of verapamil decreased in all three SVT groups (*P* < *0.05*). Use of amiodarone and class 1C antiarrhythmics remained low, with the highest usage among EAT patients.

**Conclusion:**

Between 2001 and 2018, CA was increasingly performed in patients with SVT, primarily AVNRT- and EAT patients. The burden of comorbidities increased. Patients undergoing CA without prior antiarrhythmic- or rate-limiting drug therapy increased significantly. Use of beta-blockers increased and remained the most widely used drug.

**Graphical Abstract:**

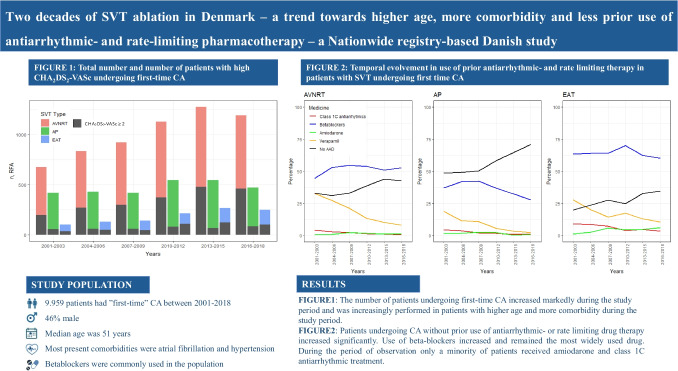

**Supplementary Information:**

The online version contains supplementary material available at 10.1007/s10840-023-01692-9.

## Introduction

Supraventricular tachycardias (SVTs) are a common cause of presentation to hospital care services. The term “SVT” indicates tachycardia [atrial rates > 100 beats per minute (b.p.m.) at rest], the mechanism of which involves tissue from the HIS bundle or above.[1].

SVTs are rarely life-threatening, but some individuals experience severe symptoms such as syncope, and, though unusual, SVT can sometimes lead to cardiac arrest. Symptom burden differ from patient to patient and can, especially in chronic cases, have a great impact on the patient’s quality of life with for example reduced ability to go to work or exercise. [2, 3].

Treatment options range from none to vagal maneuvers, antiarrhythmic drug therapy (AAD), and long-term management with catheter ablations.[4] Over the last decades, catheter ablation (CA) has emerged with improved techniques, making it a feasible treatment option even for older and more comorbid patients. According to current guidelines from the European Society of Cardiology, CA should be considered first-line therapy for all symptomatic SVT patients. [5] However, little is known about how treatment selection and comorbidity burden has changed over time in this population. Further, though CA is considered curative in most patients, studies regarding use of antiarrhythmic- and rate limiting medication prior to catheter ablation are limited.

Using the nationwide Danish registries, this project aimed to describe the evolution in the use of catheter ablation for SVT patients and changes in characteristics of patients undergoing first-time ablation for either AVNRT, AP, or EAT in Denmark between 2001 and 2018. Further, as CA has been implemented as treatment option and recommended as first-line therapy, we aimed to investigate changes in the use of AAD in the population prior to CA.

## Methods

### Data sources

In this register-based study, information regarding procedures, comorbidities, and medication was identified using nationwide Danish registers. The Danish National Patient Register (DNPR) contains information on all hospital admissions in Denmark. At discharge, all admissions are encoded with a primary diagnosis and commonly secondary diagnoses, according to the International Classification of Disease, 10^th^ revision (ICD-10). DNPR also holds information on operations and procedures, including catheter ablations. [6, 7].

The Danish National Prescription Registry (DNPR) keeps records of all redeemed prescriptions coded according to the Anatomical Therapeutic Chemical (ATC) Classification System. For each medication dispensed, the register also contains information about date of dispensing, quantity, strength, and composition of medicines. [8] Data from the registers were cross-linked using the unique personal identification number from the Civil Registration system, which contains data on age, sex, and vital status of all residents in Denmark. The system assigns a unique personal identification number to all Danish citizens at the time of birth or date of immigration.[9].

### Study population

All patients who underwent a first-time CA for AVNRT, AP, or EAT in Denmark between January 1, 2001, and December 31, 2018, were identified with operation codes. (Supplementary Table 1). Patients aged 18 years or above at the time of their first ablation were included, with inclusion at procedure date. Exclusion criteria were patients with history of previous ablation for atrial fibrillation or atrial flutter or patients not residing in Denmark.

The population was divided into three subgroups by the type of SVT: AVNRT, AP, and EAT patients. Temporal trends were examined from 2001 to 2018 by three-year intervals.

### Comorbidities and medications

Comorbidities were identified 5 years prior to ablation from DNPR including ischemic heart disease, heart failure, hypertension, atrial fibrillation/-flutter, and diabetes. Hypertension was defined as the use of at least two different anti-hypertensive drugs. Diabetes was defined by use of diabetes medication both insulin and non-insulin treatments. Use of beta-blockers, verapamil, class 1C antiarrhythmics, and amiodarone 2 years prior to ablation was identified from the Danish National Prescription Registry (Supplementary Table 1).

### Patient characteristics

Patient characteristics were identified at the date of procedure and presented in two groups by CHA_2_DS_2_-VASc (congestive heart failure, hypertension, age > 75, diabetes, stroke, vascular disease, age 65 to 74 and female sex) score < 2 or ≥ 2, with a high score indicating higher age and more comorbidity. The CHA_2_DS_2_-VASc scale is a point-based system originally developed to stratify the risk of stroke and systemic embolism in patients with atrial fibrillation. [10, 11].

### Statistical analysis

Baseline tables were employed to describe the study population at the ablation date. Continuous variables were reported by the median and inter-quartile range (IQR), and categorical variables were summarized with counts and percentages. Multivariable logistic regression models were applied to identify covariates associated with use of antiarrhythmic- and rate limiting medication. The models were adjusted for calendar year, age (< 65 as reference), sex (women as reference), hypertension, heart failure, and ischemic heart disease. All statistical calculations were performed using R (R Core Team (2015). R: A language and environment for statistical computing. R Foundation for Statistical Computing, Vienna, Austria. URL http://www.R-project.org/.)

### Ethics

Approval for register-based studies is not required from an ethics committee under Danish law. The Danish Data Protection Agency approved this study (Approval number: P-2019–404).

## Results

### Population

A total of 11,860 patients underwent first-time RFA between January 1, 2001, and December 31, 2018, of which 9959 patients met the criteria for inclusion in the study cohort. A total of 6023 (61%) received RFA for AVNRT, 2829 (28%) for AP, and 1107 (11%) for EAT. Overall, 46% of the study population were male with a distribution of 39%, 61%, and 43% in the AVNRT, AP, and EAT group, respectively. The overall median age and interquartile range [IQR] was 51 years [37–63] ranging from 42 [29, 55], 55 [42–65], to 55 [41–66] for AP, AVNRT, and AP patients, respectively.

The most prevalent comorbidities were hypertension (28%), atrial fibrillation (18%), and ischemic heart disease (10%). The morbidity burden was generally higher in patients treated for EAT. Markedly, atrial fibrillation was diagnosed in 36% of EAT patients. Beta-blockers were the most widely used antiarrhythmic- and rate-limiting drugs. Only 2% used class 1C antiarrhythmics amiodarone up to 2 years prior to ablation. The use of AAD was highest in the EAT group (Table 1).

**Table 1:** Baseline characteristics of the study population

	Overall	AVNRT	AP	EAT
*n*	9959	6023	2829	1107
Age (median (IQR))	51 [37, 63]	55 [42, 65]	42 [29, 55]	55 [41, 66]
Male (%)	46%	39%	61%	43%
Comorbidities (%)				
Atrial fibrillation	1829 (18)	1071 (18)	362 (13)	396 (36)
Syncope	479 (5)	302 (5)	120 (4)	73 (5)
Heart failure	347 (4)	193 (3)	55 (2)	99 (9)
Ischemic heart disease	1021 (10)	649 (11)	215 (8)	157 (14)
Valvular heart disease	183 (2)	124 (2)	25 (1)	53 (4)
Hypertension	2769 (28)	1882 (31)	437 (15)	450 (41)
Chronic obstructive pulmonary disease	298 (3)	207 (3)	49 (2)	42 (4)
Diabetes mellitus	481 (5)	343 (6)	72 (3)	66 (6)
Stroke	210 (2)	138 (2)	41 (1)	31 (3)
CHA_2_DS_2_-VASc ≥ 2	2950 (30)	2084 (35)	406 (14)	460 (42)
Medication (%)				
Beta-blockers	4851 (49)	3127 (52)	1017 (36)	707 (64)
Verapamil	1409 (14)	1010 (17)	228 (8)	171 (15)
Digoxin	269 (3)	145 (2)	44 (2)	80 (7)
Amiodarone	147 (2)	65 (1)	35 (1)	47 (4)
Class 1C antiarrhythmics	217 (2)	100 (2)	59 (2)	58 (5)
All calcium channel inhibitors	2216 (22)	1544 (26)	378 (13)	294 (27)
Renin-angiotensin inhibitors	1822 (18)	1212 (20)	313 (11)	297 (27)
Diuretics	1833 (18)	1237 (21)	296 (11)	300 (27)
Oral anti-coagulants	903 (9)	518 (9)	120 (4)	265 (24)

### Evolution of CA use during the study period

**Fig. 1:**
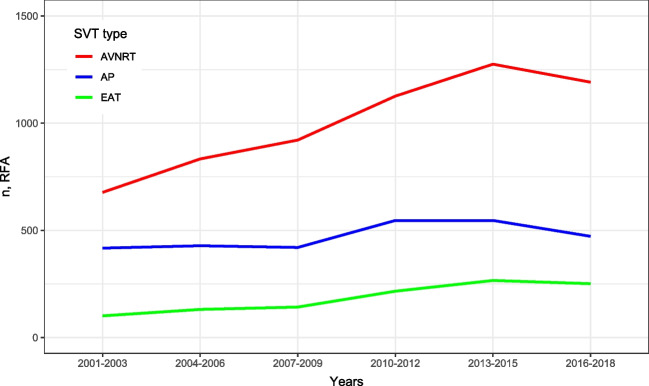
Evolvement in number of patients with SVT undergoing first time CA. *X* axis depicts time. *Y* axis depicts number of CAs

Figure 1 depicts the evolution of the number of patients with SVT undergoing first-time CA between 2001 and 2018. During the study period, the number of patients receiving CA increased nearly 40% from 1195 in 2001–2003 to 1914 in 2016–2018. This increase was especially pronounced among AVNRT and EAT patients. Numbers are listed in Table 2.

**Table 2:** Percentage of patients undergoing first-time CA with CHA_2_DS_2_-VASc ≥ 2 during the study period

	2001–2003	2004–2006	2007–2009	2010–2012	2013–2015	2016–2018	*P* value
AVNRT							
Total (%)	677 (100)	833 (100)	921 (100)	1126 (100)	1275 (100)	1191 (100)	
CHA_2_DS_2_-VASc ≥ 2 (%)	198 (29)	272 (33)	300 (33)	372 (33)	480 (38)	462 (39)	*P* < *0.05*
AP							
Total (%)	417 (100)	428 (100)	420 (100)	546 (100)	546 (100)	472 (100)	
CHA_2_DS_2_-VASc ≥ 2 (%)	56 (13)	60 (14)	60 (14)	79 (15)	66 (12)	85 (18)	*P* = *0.06*
EAT							
Total (%)	101 (100)	131 (100)	142 (100)	216 (100)	266 (100)	251 (100)	
CHA_2_DS_2_-VASc ≥ 2 (%)	34 (34)	49 (37)	45 (32)	110 (51)	122 (46)	100 (40)	*P* = *0.28*

### Trends in patient characteristics

**Fig. 2:**
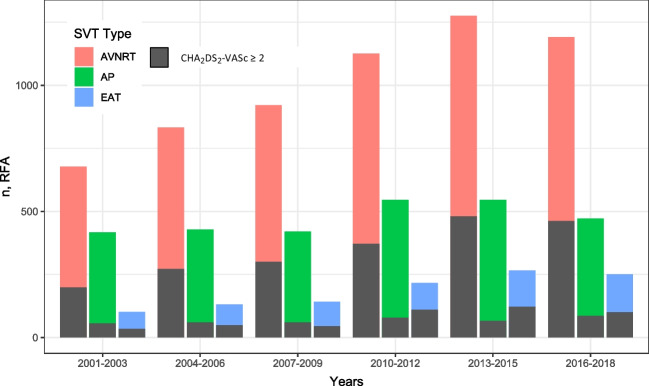
Total number and number of patients with high CHA_2_DS_2_-VASc undergoing first-time CA. *X* axis depicts time. *Y* axis depicts number of CAs

Figure 2 demonstrates that CA was increasingly performed in patients with a higher comorbidity of CHA_2_DS_2_-VASc ≥ 2 during the study period. The percentage of AVNRT patients with CHA_2_DS_2_-VASc ≥ 2 increased significantly from 30% in 2001–2003 to 40% in 2016–2018 (*P* < 0.05). In AP patients, the increase was less pronounced, from 13% in 2001–2003 to 18% in 2016–2018 (*P* = 0.06). Among EAT patients, the 3-year annual increase was 34% in 2001–2003 to 40% in 2016–2018 (*P* = 0.28) (*P* values are listed in Table 2).

### Trends in use of antiarrhythmic- and rate-limiting drugs

**Fig. 3:**
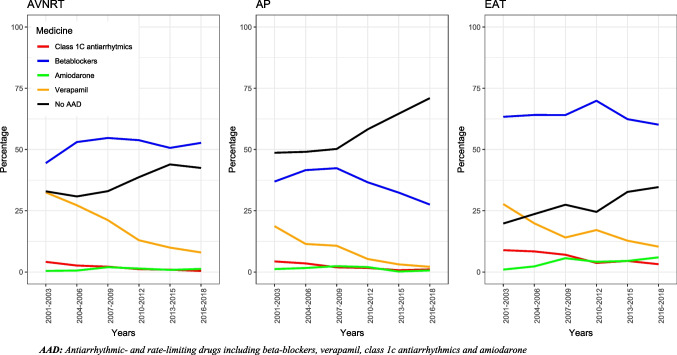
Temporal evolvement in use of prior antiarrhythmic- and rate-limiting therapy in patients with SVT undergoing first time CA. *X* axis depicts time. *Y* axis relative number of patients using antiarrhythmic- or rate-limiting medicine CAs

Figure 3* and *Table 3 depict the temporal evolution in prior antiarrhythmic- and rate-limiting therapy in patients with SVT undergoing first-time CA. The number of patients who had no prior use of antiarrhythmic- and rate-limiting therapy increased significantly among all subgroups. Notably, the increase was most pronounced in individuals with AP from 49 to 71% *(P* < *0.05)* (Table 3).

**Table 3:** *P* values. Temporal evolvement in use of prior antiarrhythmic- and rate-limiting therapy in patients with SVT undergoing first time CA

SVT type	Medicine	Year (%)		*P* value
		2001–2003	2016–2018	
**AVNRT**				
	Beta-blockers	45	53	*P* < *0.05*
	Verapamil	33	8	*P* < *0.05*
	Class 1C antiarrhythmics	4	0	*P* < *0.05*
	Amiodarone	0	1	*P* = *0.08*
	No prior AAD	33	43	*P* < *0.05*
AP				
	Beta-blockers	38	28	*P* < *0.05*
	Verapamil	19	2	*P* < *0.05*
	Class 1C antiarrhythmics	4	1	*P* < *0.05*
	Amiodarone	1	1	*P* = *0.37*
	No prior AAD	49	71	*P* < *0.05*
EAT				
	Beta-blockers	63	60	*P* = *0.58*
	Verapamil	28	10	*P* < *0.05*
	Class 1C antiarrhythmics	9	3	*P* < *0.05*
	Amiodarone	1	6	*P* < *0.05*
	No prior AAD	20	35	*P* < *0.05*
Total				
	Beta-blockers	43	47	*P* < *0.05*
	Verapamil	27	7	*P* < *0.05*
	Class 1C antiarrhythmics	5	1	*P* < *0.05*
	Amiodarone	1	2	*P* < *0.05*
	No prior AAD	37	48	*P* < *0.05*

*AAD* antiarrhythmic- and rate-limiting drugs including beta-blockers, verapamil, class 1c antiarrhythmics, and amiodarone

Beta-blockers were the most frequently used medication for all three patient groups throughout the study period. Use of beta-blockers increased from 45 to 53% (*P* < *0.05*) for AVNRT patients, decreased among AP patients from 38 to 28% (*P* < *0.05*), and remained constant for EAT patients, with 53% in 2001–2003 to 60% in 2016–2018 (*P* = *0.58*). The time trends showed a major, significant decrease in the use of verapamil towards a minor use for all SVT groups (Fig. 3 and Table 3). In general, prior use of amiodarone and class 1C antiarrhythmics remained low.

**Fig. 4:**
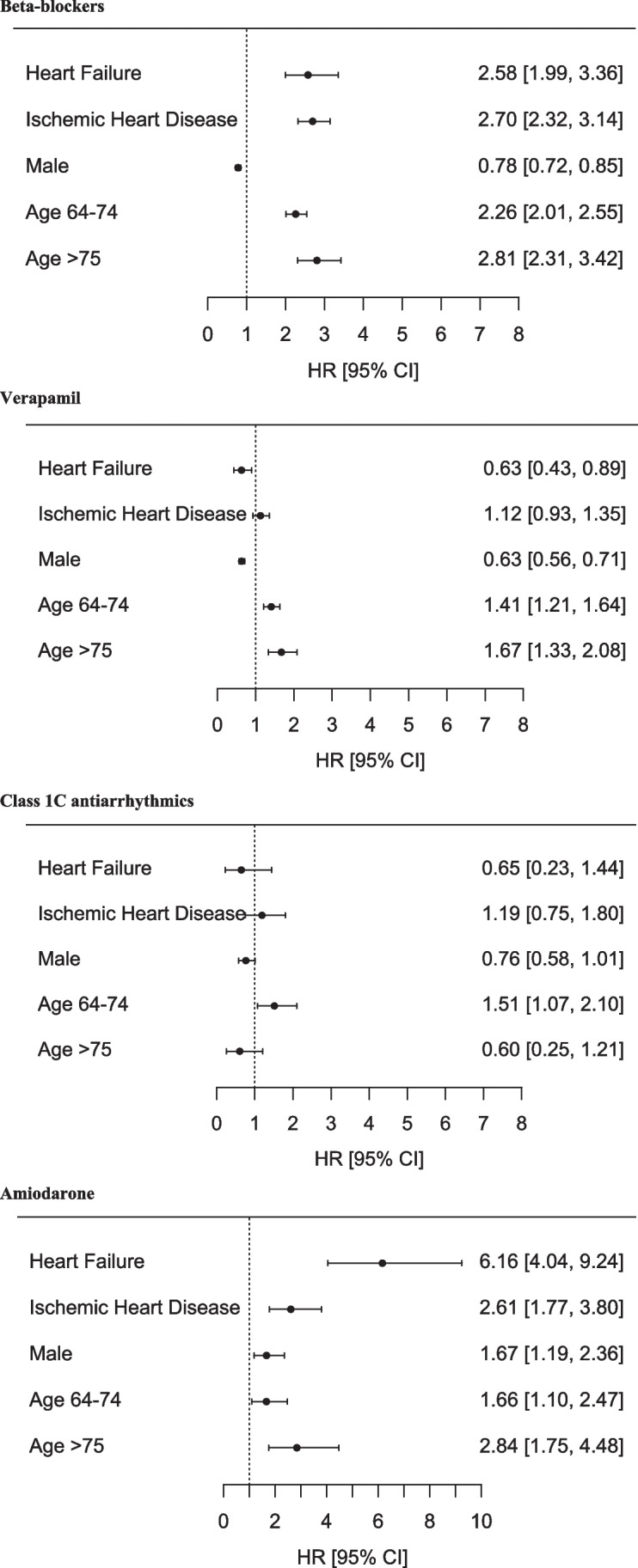
Multiple logistic regression analysis of antiarrhythmic- and rate-limiting medicine received within 2 years prior to CA. The models were adjusted for age, sex, hypertension, heart failure, and ischemic heart disease

Figure 4 depicts multivariable logistic regression analysis applied to identify covariates associated with antiarrhythmic- and rate-limiting medication use. Beta-blockers were more frequently used among patients with ischemic heart disease, female gender, and higher age. Notably, the use of amiodarone was associated with presence of heart failure and ischemic heart disease, male gender, and higher age. The presence of heart failure and higher age was not associated with use of class 1C antiarrhythmics.

## Discussion

This study has three main findings: (i) Over the last two decades, there has been a great increase in patients undergoing CA for the treatment of SVT; (ii) CA was increasingly performed in older and more comorbid patients; and (iii) patients undergoing CA without prior use of antiarrhythmic- or rate-limiting drug therapy increased significantly. Use of beta-blockers increased and remained the most widely used drug. During the period of observation, only a minority of patients received amiodarone and class 1C antiarrhythmic treatment.

During the study period, the number of patients undergoing first-time CA treatment increased markedly. This increase was especially dominant among AVNRT patients that, according to previous studies, is the second most frequent CA-treated SVT after atrial fibrillation.[12] A likely explanation for the increasing use of CA is that the capacity has increased markedly, as CA has been recommended as first-line therapy since 2003 according to ESC Guidelines.[13] The potential cure for symptoms, improved quality of life, reduction of hospital admissions, and lower cost burden both on an individual- and societal-level make CA a desirable option as first-line therapy for all types of SVT patients with chronic symptoms.[14] Previous studies have shown high success rates and low risk of recurrence, complications, and mortality in younger patients receiving CA treatment. However, recent change of pattern in patient selection due to the increased focus on CA as rhythm management and improvements in procedure techniques might affect these outcomes.[15] Our study showed that the number of patients undergoing first-time CA with CHA_2_DS_2_-VASc-score ≥ 2 increased markedly among AVNRT and EAT patients during the study period. Among the three subgroups, EAT patients were the oldest, with more comorbidity, and had the highest use of medication. Notably, EAT patients do have a higher age of SVT onset, which naturally increases CHA_2_DS_2_-VASc-score; however, it does not explain the increase over time.[5] Especially, atrial fibrillation was a frequent comorbidity among EAT patients, indicating a possible co-existence between EAT and atrial fibrillation. It cannot be ruled out that misclassification of atrial fibrillation and EAT in the clinic affects these results; however, previous studies have also shown a close association between atrial fibrillation and EAT.[16, 17] A possible explanation for the increasing age could be that younger patients compared to older patients tend to opt out CA due to the risk of severe complications as AV blockage and need for pacemaker after the procedure. However, our results reenforces the encouraging trend that ablation in clinical practice is considered an effective treatment regardless of age and comorbidity.

Importantly, our study found a significant increase in the number of patients, who underwent AP without any prior use of antiarrhythmic- or rate-limiting medicine among all three patient groups. These results and the increasing number of CAs support that more patients choose CA as first-line therapy today, as recommended in recent guidelines.[3] Our results show that beta-blocker treatment remained the “treatment of choice” for SVT patients prior to ablation. Overall, every other SVT patient undergoing first time ablation used beta-blockers at baseline. Notably, use of beta-blocker remained high and increased among AVNRT patients. This might reflect the solid documentation of beta-blocker treatment in SVT patients, and it might also reflect “a carry-over” effect from randomized trials and guidelines of other important cardiovascular diseases such as atrial fibrillation.[11, 18, 19] The trend towards use of beta-blockers might explain the observed decrease in use of verapamil prior to CA treatment, although verapamil is considered substitutable with beta-blockers in the treatment of SVT. Previous studies have demonstrated similar patterns regarding pharmacological therapy among Danish patients with atrial fibrillation.[20] Our results might highlight that clinical practice has been implemented before recent recommendations, where verapamil is recommended as second choice of treatment if ablation is not feasible.[3] During the period of observation, only a minority of patients received amiodarone and class 1C antiarrhythmic treatment prior to ablation, though these medicaments are effective to maintain sinus rhythm. The uncommon use might be because of their potential serious side effects and because prescriptions require outpatient controls that are time-consuming for the patient. Furthermore, class 1C antiarrhythmics are restricted to patients without established ischemic heart disease or left ventricular dysfunction, which limits their use.[11] Importantly, this study confirmed that Class IC antiarrhythmics were not associated with use in patients with ischemic heart disease and heart failure.

Due to study limitations, guidelines for SVT cannot make recommendations based on patient-reported quality of life.[2] Though, cost of ablation can often be counterbalanced by high long-term effectiveness and improvement in quality of life for patients with frequent episodes. Drug therapy is often characterized by long-term expense for the patient, variable patient adherence, and poor patient-satisfaction in the long-term.[2] A randomized controlled trial found drug-therapy to be less effective and not tolerated by a substantial number of symptomatic patients and an EHRA consensus document found the value of antiarrhythmic drug therapy limited in the view of the excellent success rate and minimal risk of catheter ablation.[2, 18] Though catheter ablation has been extensively evaluated in specific subgroups and in controlled trials, less is known about the real-world outcome in larger multicentric cohorts.[21] Especially, more studies on an aging and multimorbid patient group would give important information regarding success rates and risk of complications among these patients.

### Strengths and limitations

The registries used for this study comprise all patients who have undergone first-time CA treatment in Denmark, minimizing selection bias. Therefore, data in this study reflects real-world clinical practice on a nationwide scale. However, the registries do not include information regarding frequency of SVT episodes or severity of symptoms among the patients. Nor do they have information on procedure details or reason for referral. Registries do not contain information on important life-style parameters as smoking state, body mass index, and physical activity nor is detailed background information on the patients aside from age and gender available. Further, the high presentation of atrial fibrillation among the patients might be due to misclassification in the registries. Residual confounding might be present, for instance, through lack of data on blood pressure measurements and blood glucose levels that could result in underdiagnosing hypertension or diabetes mellitus affecting baseline results as well as the calculated CHA_2_DS_2_-VASc-score.

## Conclusion

In this large nationwide study, the use of CA for SVT increased considerably during a 20-year observation period. Over time, CA was increasingly utilized and performed in older patients with higher comorbidity. Interestingly, patients undergoing CA without prior use of antiarrhythmic- or rate-limiting drug therapy increased significantly. Use of beta-blockers increased and remained the most widely used drug. During the period of observation only, a minority of patients received amiodarone and class 1C antiarrhythmic treatment.

### Supplementary Information

Below is the link to the electronic supplementary material.

Supplementary file1 (DOCX 26 KB)

## Data Availability

Data on prescribed medication can, by the Danish Pharmacy Law, only be made available for research on servers hosted in highly protected research environments present in either Statistics Denmark or the Serum Institute. In these institutions, investigators can be granted permission to use the data with encrypted person identification. If other parties wish to work with the raw data, they will need to get access through collaboration with us or another institution which has been granted access. Please contact Charlotte Middelfart (charlotte.middelfart@regionh.dk) with any questions regarding data access.
