# Supplementary material for: Two decades of SVT ablation in Denmark: a trend towards higher age, more comorbidity, and less prior use of antiarrhythmic and rate-limiting pharmacotherapy—a nationwide registry-based Danish study
Source: J Interv Card Electrophysiol. 2023 Dec 18;67(4):837–46. doi: 10.1007/s10840-023-01692-9 (PMC11166800; doi:10.1007/s10840-023-01692-9)
Supplement: Supplementary file 1 — Supplementary file1 (DOCX 26 KB) [file 10840_2023_1692_MOESM1_ESM.docx]

**Supplementary Table 1**

Table S1: List of ICD-10 diagnoses codes and ATC codes

| Procedure codes for catheter ablation |  |  |
| --- | --- | --- |
| *CA for atrioventricular nodal re-entry tachycardia* | *Defined from operation codes* | SKS: BFFB20, BFFB21 |
| *CA for accessory pathways* | *Defined from operation codes* | SKS: BFFB10, BFFB11, BFFB12 |
| *CA for Ectopic atrial tachycardia* | *Defined from operation codes* | SKS: BFFB01 |
|  |  |  |
| Comorbidities  *(A+B diagnoses, patient type= 0 and 2: Hospitalization and outpatient visit)* | | |
|  | | |
| *Atrial fibrillation (AFLI)* | *Defined from diagnosis* | ICD-10: DI48 |
| *Previous ablation for AFLI* | *Defined from operation codes* | SKS: BFFB03, BFFB04 |
| *Ischemic heart disease* | *Defined from diagnosis* | ICD-10: DI20-25 |
| *Chronic heart failure* | *Defined from diagnosis* | ICD-10: DI110, DI42, DI50, DJ81 |
| *Hypertension* | *Defined from combination treatment with at least two classes of antihypertensive drugs: adrenergic α-antagonists, non-loop diuretics, vasodilators, beta-blockers, calcium channel blockers and renin-angiotension system* | *ATC: C02A, C02B, C02C, C02L,*  *C03A, C03B, C03D, C03E, C03X,*  *C07B, C07C, C07D, C08G, C02DA, C09BA, C09DA, C02DB, C02DD, C02DG, C07A, C07B, C07C, C07D, C07F, C08, C09BB, C09DB, C09AA, C09BA, C09BB, C09CA, C09DA, C09DB, C09XA02, C09XA52* |
| *Chronic obstructive pulmonary disease* | *Defined from diagnosis* | ICD-10: DJ42, DJ43, DJ44 |
| *Diabetes mellitus* | Diabetes mellitus defined from glucose lowering drugs | ATC: A10 |
| *Stroke* | *Defined from diagnosis* | ICD-10: DI63, DI64, G458, G459, I74, DI60, DI61, DI62 |

Medicine

| *ADP-receptor blockers* | Defined from ATC-codes | B01AC04, B01AC24, B01AC22 |
| --- | --- | --- |
|  |  |  |
| *Loop diuretics* | Defined from ATC-codes | C03CA01, C03EB, C03CA02 |
| *Non-loop diuretics* | Defined from ATC-codes | C02DA, C02L, C07D, C09XA52, C03A, C03EA, C03B, C03X, C07C, C08G, C09BA, C09DA, C03D, C03E |
| *Beta- blockers* | Defined from ATC-codes | C07A, C07B, C07C, C07D, C07F |
|  |  |  |
|  |  |  |
| *Calcium channel inhibitors* | Defined from ATC-codes | C08, C09BB, C09DB |
| *Renin-angiotensin system inhibitors* | Defined from ATC-codes | C09AA, C09BA, C09BB, C09CA, C09DA, C09DB, C09XA02, C09XA52 |
| *Oral anticoagulantia* | Defined from ATC-codes | B01AA03, B01AA04, B01AE07, B01AF01, B01AF02, B01AF03 |
| *Amiodarone* | Defined from ATC-codes | C01BD01 |
| *Dronedarone* | Defined from ATC-codes | C01BD07 |
| *Class 1C antiarrhythmics* | Defined from ATC-codes | C01BC |
| *Digoxin* | Defined from ATC-codes | C01AA |


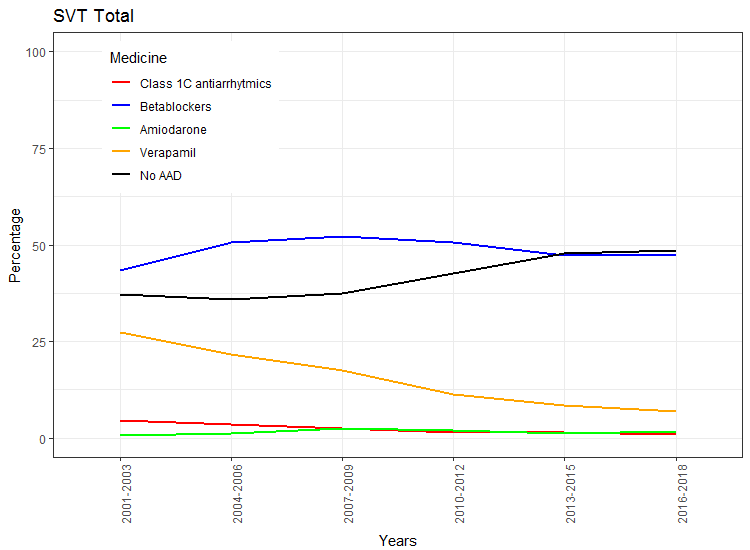
**Supplementary Figure 1: Temporal evolvement in use of prior antiarrhythmic- and rate limiting therapy in patients with SVT undergoing first time CA. X axis depicts time. Y axis depicts the relative number of patients using antiarrhythmic- or rate-limiting medicine.**

***AAD:*** *Antiarrhythmic- and rate-limiting drugs including beta-blockers, verapamil, class 1c antiarrhythmics and amiodarone*
